# Mechano-chemo-biological model of atherosclerosis formation based on the outside-in theory

**DOI:** 10.1007/s10237-023-01790-7

**Published:** 2023-12-23

**Authors:** Meike Gierig, Alexandros Tragoudas, Axel Haverich, Peter Wriggers

**Affiliations:** 1 Institute of Continuum Mechanics, Leibniz University of Hannover, An der Universität 1, 30823 Garbsen, Germany; 2 Department of Cardiothoracic, Transplantation, and Vascular Surgery, Hannover Medical School (MHH), Carl-Neuberg-Straße 1, 30625 Hannover, Germany

**Keywords:** Atherosclerosis, Outside-in theory, Mechanobiology, Finite element method

## Abstract

Atherosclerosis is a disease in blood vessels that often results in plaque formation and lumen narrowing. It is an inflammatory response of the tissue caused by disruptions in the vessel wall nourishment. Blood vessels are nourished by nutrients originating from the blood of the lumen. In medium-sized and larger vessels, nutrients are additionally provided from outside through a network of capillaries called vasa vasorum. It has recently been hypothesized (Haverich in Circulation 135:205–207, 2017) that the root of atherosclerotic diseases is the malfunction of the vasa vasorum. This, so-called outside-in theory, is supported by a recently developed numerical model (Soleimani et al. in Arch Comput Methods Eng 28:4263–4282, 2021) accounting for the inflammation initiation in the adventitial layer of the blood vessel. Building on the previous findings, this work proposes an extended material model for atherosclerosis formation that is based on the outside-in theory. Beside the description of growth kinematics and nutrient diffusion, the roles of monocytes, macrophages, foam cells, smooth muscle cells and collagen are accounted for in a nonlinear continuum mechanics framework. Cells are activated due to a lack of vessel wall nourishment and proliferate, migrate, differentiate and synthesize collagen, leading to the formation of a plaque. Numerical studies show that the onset of atherosclerosis can qualitatively be reproduced and back the new theory.

## Introduction

**Fig. 1:**
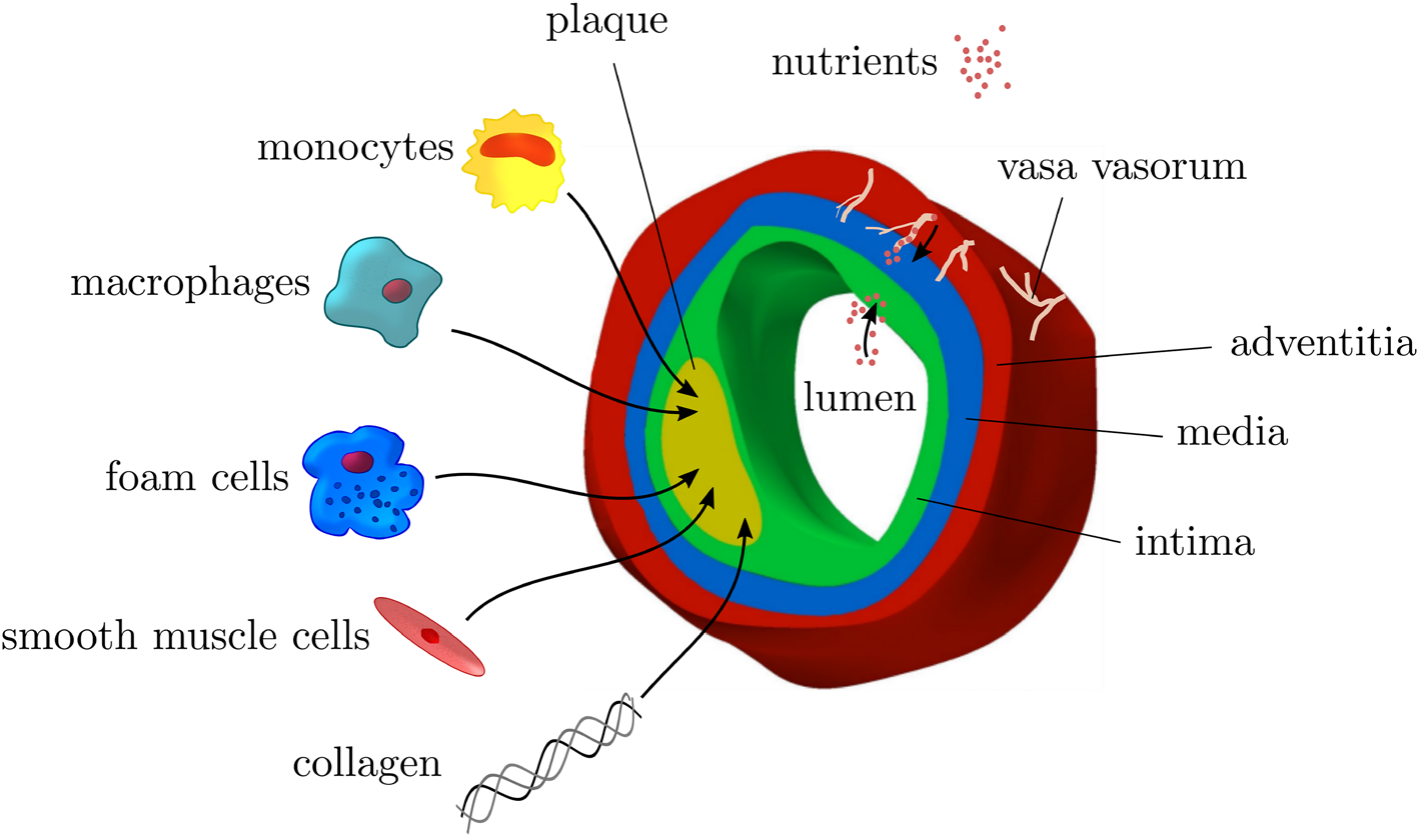
layers of an artery, composition of a plaque and nutrient supply from the lumen and through vasa vasorum

Atherosclerosis is a pathology in medium and large-sized arteries such as cerebral, coronary and peripheral arteries (Postiglione and Napoli 1995; Hansson 2005; Hussein et al. 2011), and the most common cause of cardiovascular disease (Wilkins et al. 2017). A major manifestation of atherosclerosis is the obstruction of the vessel lumen by plaques, consisting of calcified connective tissue and cholesterol, resulting in turn from the excessive accumulation of cells, debris and extracellular matrix constituents (Weber and Noels 2011), Fig. 1.

In the onset of atherosclerosis, inflammatory events take place (Spagnoli et al. 2007) and cellular as well as molecular species accumulate. As a response to inflammation, immune cells such as monocytes and T-cells enter the vessel wall, the latter species secreting interferon gamma (IFN-$\gamma$) (Hansson et al. 1989). Monocytes are attracted by monocyte chemoattractant protein-1 (MCP-1) at the site of inflammation where they are stimulated by interferon gamma (IFN-$\gamma$) to differentiate into macrophages (Weber and Noels 2011). Beside the immune cells, low-density-lipoproteins (LDL) are recruited to the site of inflammation and become oxidized contacting with free oxygen radicals (Yoshida and Kisugi 2010). These oxidized LDL contain a high amount of cholesterol and are consumed by macrophages (phagocytosis) that turn into foam cells (Gui et al. 2012). These in turn undergo apoptosis and secrete MCP-1 and platelet-derived growth factors (PDGF) what attracts monocytes and smooth muscle cells, respectively. As a consequence, also smooth muscle cells (SMC) accumulate at the site of inflammation and synthesize collagen. These mechanisms cause the formation of a plaque, consisting mainly of monocytes, macrophages, smooth muscle cells, foam cells, collagen and the cholesterol-rich debris of foam cells.

Although the pathways of atherosclerosis are understood in much detail, the initial pathogenetic event remains unclear. Two theories exist that are outlined in the following.

In the first one, as by Ross (1993), injury of the endothelium and intima due to mechanical damage, toxins or oxygen radicals is assumed to initiate inflammation. Due to the impaired endothelium, LDL and monocytes diffuse into the intima and the chain of event takes its course. Atherosclerosis is hence a pathology that starts in the intima and develops further toward the media and adventitia. This perception is consequently referred to as *inside-out-theory*. Often, high wall shear stress due to blood flow is named as source of damage (Cunningham and Gotlieb 2005; Gimbrone Jr and García-Cardeña 2013).

In contrast, observations reported by Haverich (2017) promote an *outside-in theory*. Haverich observed that atherosclerosis predominantly evolves in arteries that are supplied through a vasa vasorum (VV) network in the adventitia, i.e., through microvessels, Fig. 1. In his theory, the inflammatory event is initiated due to VV obstruction or disruption and hence impaired nutrient supply. The subsequent arterial wall ischemia represents an early pathophysiological mechanism in the onset of atherosclerosis. The major proposition by Haverich (2017) is consequently that atherosclerosis is a microvascular disease and larger arteries are involved secondarily after microvascular disease of their vessel wall. Atherosclerosis is hence initiated in the adventitia and propagates inwards toward media and intima.

In the literature, numerical approaches to model atherosclerosis are mainly based on the inside-out-theory. Several studies focus on the role of LDL in the arterial wall (Cobbold et al. 2002; Hao and Friedman 2014) or in both, the blood stream and arterial wall (Olgac et al. 2008; Dabagh et al. 2009; Di Tomaso et al. 2011; Cilla et al. 2014) to additionally consider the role of blood flow dynamics. Some authors present detailed chemo-biological models such as (Hao and Friedman 2014) accounting for several lipoproteins, free radicals, matrix metalloproteinases and their inhibitors, cytokines, macrophages, foam cells, T-cells and smooth muscle cells. Cilla et al. (2014) takes into account LDL, oxidized LDL, monocytes, macrophages, foam cells, smooth muscle cells, cytokines and collagen. However, a consistent description of growth kinematics is missing in these publications and mechanical aspects such as homeostasis are not incorporated. A detailed review of models based on the inside-out-theory can be found in Parton et al. (2016).

A first modeling approach dedicated to the outside-in theory was proposed by Soleimani et al. (2021). In their model, growth is driven by inflammation, which is initiated in the middle layer of the artery, where a VV obstruction would cause nutrient scarcity. Employing a phase-field approach, inflammation propagates inwards along a gradient of nutrient concentration. While growth kinematics is well defined, the model does not account for biological species that drive plaque development.

The present work proposes a continuum mechanical description featuring the outside-in theory (Haverich 2017) of atherosclerosis. It combines a thorough continuum mechanical description as in Soleimani et al. (2021) with a great detail of important chemo-biological processes as in Cilla et al. (2014) to describe atherosclerosis initiation and plaque formation. Occlusions of the vasa vasorum network are the initiators of inflammatory events. More precisely, nutrient diffusion from the lumen and VV is employed via diffusion–reaction equations and a nutrient scarcity initiates the process of atherosclerosis. A biological model part takes into account the contributions of cells to plaque formation, considering monocytes, macrophages, foam cells and smooth muscle cells. In addition, collagen synthesis from smooth muscle cells is incorporated and both, growth and remodeling, are accounted for in the context of nonlinear continuum mechanics. While the growth description governs the plaque development, remodeling accounts for the turnover of constituents and ensures the adaption of the tissue to changed loading conditions.

## Methods

**Fig. 2:**
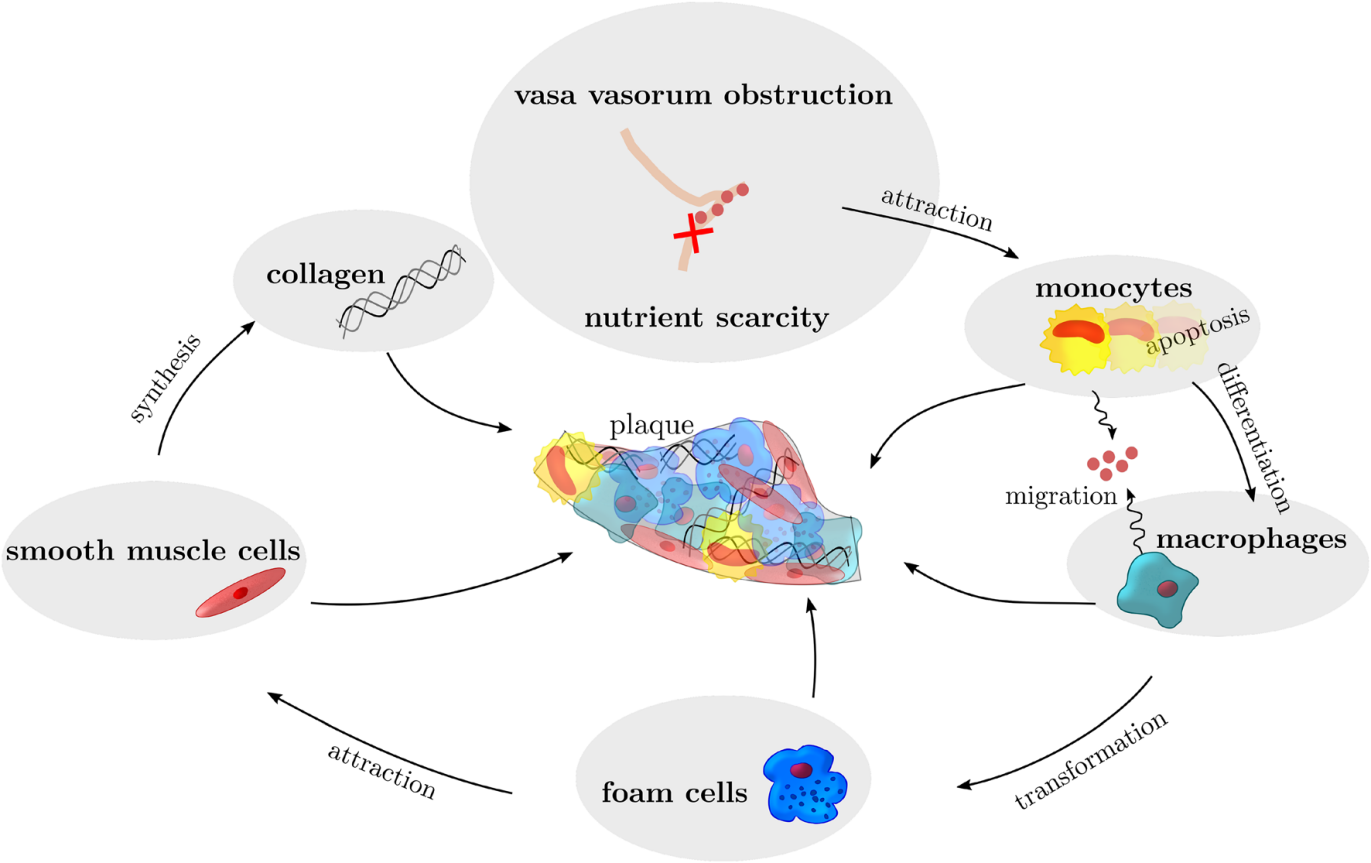
model assumptions of the chain of events

Atherosclerosis is modeled as coupled mechano-chemo-biological process. Both the mechanical and the chemo-biological model parts are presented in what follows, before the construction of the VV network is described.

### Mechanical model

Tissue mass is mainly made up of extracellular matrix (ECM) and cells. From the mechanical point of view, the ECM is the load-bearing structure of an artery (Burton 1954). It is modeled comprised of an isotropic elastic matrix and collagen fiber families. The isotropic matrix primarily consists of elastin which has a half-life time of decades of years (Burnett et al. 1982). Collagen is oriented in preferred directions and induces anisotropy. In contrast to the matrix, its half-life time is in the range of days to months (Nissen et al. 1978). Although growth factors and enzymes such as matrix metalloproteinases can act on several ECM components, only their influence on collagen is taken into account herein. Consequently, while the matrix is considered as purely elastic material, collagen is additionally assumed to turn over. That is, mass increments are removed and replaced by new mass, inheriting a certain prestretch (Bergel 1960) and keeping the net mass constant. In turn, changes in net mass of cells and ECM alter the tissue volume and result in plaque growth, directly linking mechanics with chemo-biology.

#### Kinematics

Tissue mechanics is described by employing continuum mechanical principles in the framework of finite deformations. The deformation gradient describes the deformation process and is a function of the gradient of displacements $\textbf{u}$

1 $$\begin{aligned} \textbf{F}= \textbf{I}+ {\nabla}_{\textbf{X}}\textbf{u}\,. \end{aligned}$$

To account for growth, elastic mechanisms, homeostasis and prestretch, the deformation gradient is multiplicatively decomposed

2 $$\begin{aligned} \begin{aligned} \textbf{F}&= \textbf{F}_{\textrm{e}}^{\text {c}j}\, \textbf{F}_{\textrm{r}}^{\text {c}j}\\&= \textbf{F}_{\textrm{e}}^{\text {m}}\,. \end{aligned} \end{aligned}$$

For the *j*-th collagen fiber family, an inelastic remodeling $\textbf{F}_{\textrm{r}}^{\text {c}j}$ and an elastic $\textbf{F}_{\textrm{e}}^{\text {c}j}$ deformation gradient are introduced. Assuming that the prestretch in collagen equals the homeostatic stretch, the remodeling deformation governs homeostasis as well as prestretch. The elastic deformation gradient accounts for the elastic deformation and volume change due to growth. The matrix material (superscript $\text {m}$) behaves purely elastically and is described by $\textbf{F}_{\textrm{e}}^{\text {m}}$.

As the deformation gradient, the determinant of the deformation gradient $J=\det \! \left( \textbf{F}\right)$ can be multiplicatively decomposed

3 $$\begin{aligned} J&= J_\textrm{e}^{\text {c}j}\underbrace{J_\textrm{r}^{\text {c}j}}_{=1} = \det \! \left( \textbf{F}_{\textrm{e}}^{\text {c}j}\right) \underbrace{\det \! \left( \textbf{F}_{\textrm{r}}^{\text {c}j}\right) }_{=1} \end{aligned}$$

4 $$\begin{aligned}&= J_\textrm{e}^{\text {m}}= \det \! \left( \textbf{F}_{\textrm{e}}^{\text {m}}\right) \, . \end{aligned}$$

Note that the remodeling process is isochoric by definition, cf. Eq. (14), such that a volume change is solely a consequence of elastic deformations.

A suitable deformation measure on the basis of the elastic deformation gradient $\textbf{F}_\textrm{e}^i$ of the *i*-th constituent is the elastic right Cauchy–Green tensor

5 $$\begin{aligned} \textbf{C}_\textrm{e}^i = \left( \textbf{F}_\textrm{e}^i \right) ^\textrm{T}\textbf{F}_\textrm{e}^i \,. \end{aligned}$$

The first invariant of the isochoric part of the right Cauchy–Green tensor $\varvec{\bar{C}}_\textrm{e}^\text {m}= \left( J_\textrm{e}^{\text {m}}\right) ^{-2/3} \textbf{C}_\textrm{e}^\text {m}$ reads

6 $$\begin{aligned} \bar{I}_{1\textrm{e}}^\text {m}= \textrm{tr}(\varvec{\bar{C}}_\textrm{e}^\text {m}) \,. \end{aligned}$$

To account for stretch in fiber direction, the invariant $I_{4\textrm{e}}^{\text {c}j}$ can be introduced which can be interpreted as the squared elastic stretch $\lambda _\text {e}^j$ in collagen fiber direction $\textbf{a}^j$. It is given by

7 $$\begin{aligned} I_{4\textrm{e}}^{\text {c}j}= \textbf{C}_\textrm{e}^{\text {c}j}\cdot \left( \textbf{a}^j\otimes \textbf{a}^j\right) \,. \end{aligned}$$

The orientation of collagen fibers for cylindrical geometries such as arteries is

8 $$\begin{aligned} \textbf{a}^j= \left[ -\cos (\theta _\textrm{el}^j) \sin (\theta _\textrm{az}^j), \cos (\theta _\textrm{el}^j) \cos (\theta _\textrm{az}^j), \sin (\theta _\textrm{el}^j) \right] \, \end{aligned}$$

with elevation and azimuth angles $\theta _\textrm{el}^j$ and $\theta _\textrm{az}^j$. The elevation angle is a material parameter that determines the deviation of the fiber orientation from the circumferential direction toward the axial one. Assuming a homogeneous fiber distribution in each tissue layer, it is constant for all corresponding material points. Furthermore, it is assumed that inelastic deformations do not affect the fiber orientations, and hence, the orientation vectors in the intermediate configurations equal the reference orientation $\textbf{a}^j$. In contrast, the azimuth angle determines the circumferential direction and thus depends on the position of a material point.

#### Mechanical stresses

To approximate the stress–strain response of arterial layers, an anisotropic strain energy density function is employed

9 $$\begin{aligned}{} & {} \Psi = \rho _0^\text {m}\underbrace{\mu \left( {\bar{I}}_1^\text {m}-3\right) }_{=:W^\text {m}} + \sum _{j=1}^{n_\textrm{F}} \rho _{0}^{\text {c}j} \underbrace{\frac{k_1}{2 k_2} \left( \textrm{e}^{k_2 \langle I_{4\textrm{e}}^{\text {c}j} -1\rangle ^{2}} -1 \right) }_{=:W^{\text {c}j}} \nonumber \\{} & {} + \rho _0 \frac{\kappa }{2} \left( J - \frac{\rho _0}{\rho _0(0)} \right) ^2 \,. \end{aligned}$$

The first term accounts for the isotropic tissue response of the matrix with matrix reference mass density $\rho _0^\text {m}$ and constitutive parameter $\mu > 0$ (energy per unit mass). The second term captures the behavior of $n_\textrm{F}$ collagen fibers that induce anisotropy with collagen reference mass densities $\rho _{0}^{\text {c}j}$, parameters $k_1 > 0$ (energy per unit mass) and $k_2 > 0$ (dimensionless). The Macaulay brackets

10 $$\begin{aligned} \langle \bullet \rangle = {\left\{ \begin{array}{ll} \bullet , &{} \text {if} \; \bullet \ge 0 \\ 0, &{} \text {if} \; \bullet < 0 \end{array}\right. } \,. \end{aligned}$$

ensure that the fibers carry load only under tension (i.e., $I_{4\textrm{e}}^{\text {c}\, j} > 0$).

The last term in Eq. (9) with total reference mass density $\rho _0$ and constitutive parameter $\kappa > 0$ (energy per unit mass) relates the volume change to the change of mass and enforces incompressibility in the limit ($\kappa \rightarrow \infty$).

##### Remark 1

The definition of the last term in Eq. (9) differs from classical approaches as in Mousavi et al. (2019) since the growth direction is not prescribed (through a growth deformation gradient) but results from the stiffness. See Braeu et al. (2019) for details on the general idea behind this formulation and Gierig et al. (2021, 2023) for applications. Furthermore, as the strain energy density function is defined in Lagrangian description, parameter $\kappa$ is defined herein as energy per unit mass and multiplied by the total reference mass density. This formulation has already been used by Gierig et al. (2021, 2023) and is described in detail in the first publication. In contrast to classical approaches, mass changes cause a change of the factor with which a constant spatial mass is enforced. However, it should be noted that mapping the strain energy density to the current configuration (per unit current volume) yields an (almost constant) factor $\rho \, \kappa$ as used in classical formulations. In the opinion of the authors, the herein employed formulation is sounder in the context of continuum mechanics applied to bodies with changing mass. Furthermore, the authors noted that with increasing mass, the herein applied formulation yields a lower error in the (constant) total density. It should be noted that for sufficiently high $\kappa$ and hence an almost constant spatial mass density, different formulations of the last term in Eq. (9) would rarely affect the results.

The 1st Piola–Kirchhoff stress tensor follows as

11 $$\begin{aligned} \textbf{P}= \frac{\partial \Psi }{\partial \textbf{F}} \,. \end{aligned}$$

Bearing in mind that atherosclerosis is developed on a large time scale, inertia terms can be neglected. By further neglecting body forces, the balance of linear momentum reads

12 $$\begin{aligned} {\nabla}_{\textbf{X}}\cdot \left( \textbf{P}\right) = 0 \,. \end{aligned}$$

The Cauchy stress tensor is obtained as

13 $$\begin{aligned} \varvec{\sigma }= \frac{1}{J}\textbf{P}\, \textbf{F}^\textrm{T}\,. \end{aligned}$$

#### Tissue remodeling

In vivo, tissues inherit a stable mechanical state (homeostasis) which is characterized by certain stretch or stress levels. Such a preloading clearly influences the mechanical tissue behavior and is hence incorporated in the presented framework. As in Grytsan et al. (2017), an isochoric, inelastic deformation gradient $\textbf{F}_{\textrm{r}}^{\text {c}j}$ is introduced

14 $$\begin{aligned} \textbf{F}_{\textrm{r}}^{\text {c}j}= \lambda _\text {r}^j \left( \textbf{a}^j\otimes \textbf{a}^j\right) + \frac{1}{\sqrt{\lambda _\text {r}^j}} \left[ \textbf{I}- \left( \textbf{a}^j\otimes \textbf{a}^j\right) \right] \,. \end{aligned}$$

The remodeling stretch $\lambda _\text {r}^j$ evolves in collagen fiber direction such that a prestretch $\lambda _\text {pre}^j$ in fiber direction results. Consequently, the rate of remodeling stretch $\dot{\lambda }_\text {r}^j$ is governed by the difference between elastic tissue stretch $\lambda _\text {e}^j$ and prestretch $\lambda _\text {pre}^j$

15 $$\begin{aligned} \dot{\lambda }_\text {r}^j = k_\text {r}^j \frac{\lambda _\text {e}^j - \lambda _\text {pre}^j}{\lambda _\text {pre}^j - 1} \,, \end{aligned}$$

$k_\text {r}^j > 0$ being a parameter. Note that with this approach, homeostatic stretch and prestretch coincide.

#### Tissue growth

Cells, their debris and synthesized molecules represent a major part of plaques. Consequently, the masses of cells and collagen are chosen here to predict the plaque development. The overall cell concentration per unit reference volume is the sum of monocytes ($C_{\textrm{Mo}}$), macrophages ($C_{\textrm{Ma}}$), foam cells ($C_\textrm{F}$) and smooth muscle cells ($C_\textrm{SMC}$) concentrations

16 $$\begin{aligned} C_\textrm{cells}= C_{\textrm{Mo}}+ C_{\textrm{Ma}}+ C_\textrm{F}+ C_\textrm{SMC}\,. \end{aligned}$$

When a cell’s mass is known, the cell content can be quantified in terms of reference mass densities. Assuming a constant cell mass $\alpha$ for all cells leads to

17 $$\begin{aligned} \rho _0^\textrm{cells}&= \alpha \left( C_{\textrm{Mo}}+ C_{\textrm{Ma}}+ C_\textrm{F}+ C_\textrm{SMC}\right) \, , \end{aligned}$$

18 $$\begin{aligned} \dot{\rho }_0^\textrm{cells}&= \alpha \left( \dot{C}_{\textrm{Mo}}+ \dot{C}_{\textrm{Ma}}+ \dot{C}_\textrm{F}+ \dot{C}_\textrm{SMC}\right) \, . \end{aligned}$$

The tissue reference density is consequently the sum of matrix, collagen and cell densities

19 $$\begin{aligned} \rho _0&= \rho _0^\text {m}+ \rho _0^\text {c}+ \rho _0^\textrm{cells}\, , \end{aligned}$$

20 $$\begin{aligned} \dot{\rho }_0&= \dot{\rho }_0^\text {m}+ \dot{\rho }_0^\text {c}+ \dot{\rho }_0^\textrm{cells}\, . \end{aligned}$$

Hence, with Eq. (9) at hand and assuming an (almost) constant spatial mass density, a change in a constituent’s mass directly translates into a change of volume. The direction of growth is not explicitly defined through the introduction of a growth deformation gradient but results from the minimization of the energy potential. More precisely, growth will occur in the most compliant or least stiffest directions, see Braeu et al. (2019) for more details. What remains is the formulation of the mass balance equations for the constituents, which will be performed in Sect. 2.2. The detailed derivation is described for a similar problem in Gierig et al. (2023).

### Chemo-biological model

The second part of the model describes the pathways leading from vessel ischemia to accumulation of cells and molecules and hence to plaque development. The considered pathways and interactions are schematically illustrated in Fig. 2.

#### Nutrients

Nutrients are transported in the lumen of the blood vessel as well as in the vasa vasorum and diffuse through the vessel wall where they are consumed to nourish the tissue. Consequently, nutrient transport in the wall is modeled adopting a diffusion–reaction equation for nutrient concentration $C_{\textrm{N}}$ that reads in the reference configuration

21 $$\begin{aligned}{\nabla}_{\textbf{X}}\cdot \left( D_{\text {N}}J \textbf{C}^{-1}{\nabla}_{\textbf{X}}\left( C_{\textrm{N}}\right) \right) - R_\text {N}= 0 \,. \end{aligned}$$

The reaction term $R_\text {N}> 0$ is assumed to be constant, i.e., nutrients are consumed at a constant rate. Assuming that nutrient migration speed reduces with increasing plaque size, the diffusion coefficient of nutrients $D_{\text {N}}$ depends on the plaque size function $f_\rho \in [0,1]$

22 $$\begin{aligned} f_\rho = \frac{\left\langle \tilde{\rho }_0- \frac{\rho _0}{\rho _0(0)} \right\rangle }{\tilde{\rho }_0-1} \,, \end{aligned}$$

where $\tilde{\rho }_0$ is the total mass ratio at which minimum diffusion speed is reached and $\langle \bullet \rangle$ are Macaulay brackets. The diffusion coefficient is formulated as linear function of $f_\rho$

23 $$\begin{aligned} D_{\text {N}}= (1 - f_\rho ) D_{\mathrm {N_{min}}}+ f_\rho D_{\mathrm {N_{max}}}\,. \end{aligned}$$

As a result, it is highest ($D_{\mathrm {N_{max}}}$) in healthy tissue and decreases for increasing mass linearly to a value $D_{\mathrm {N_{min}}}$.

#### Monocytes

In contrast to the model presented by Soleimani et al. (2021), inflammation is not explicitly modeled herein. Instead, rather the consequence of nutrient scarcity and hence inflammation is modeled, that is, monocytes accumulate in the tissue. The governing diffusion–advection–reaction equation for the monocytes concentration $C_{\textrm{Mo}}$ is

24 $$\begin{aligned} & \dot{C}_{\textrm{Mo}}= {\nabla}_{\textbf{X}}\cdot ( \underbrace{D_{\textrm{Mo}}J \textbf{C}^{-1}{\nabla}_{\textbf{X}}C_{\textrm{Mo}}}_{\text {diffusion}} - \underbrace{r_{\textrm{Mo}}C_{\textrm{Mo}}\textbf{C}^{-1}{\nabla}_{\textbf{X}}C_{\textrm{N}}}_{\text {advection}} )\nonumber \\{} & {} - \underbrace{E_{\textrm{MoMa}}C_{\textrm{Mo}}}_{\text {differentiation}} - \underbrace{a_{\textrm{Mo}}C_{\textrm{Mo}}}_{\text {apoptosis}} \,. \end{aligned}$$

The immune cells diffuse through the tissue, with $D_{\textrm{Mo}}$ being the diffusion coefficient, and differentiate with a constant rate $E_{\textrm{MoMa}}$ toward macrophages. Furthermore, they undergo cell death which is described through the apoptosis parameter $a_{\textrm{Mo}}$. An important feature of the model is the chemotactic behavior of monocytes with $r_{\textrm{Mo}}$ being a parameter. It is assumed that they preferably migrate along the gradient of nutrient concentration toward higher nutrient concentrations. This causes a movement inwards toward the lumen, where the artery is still fully supplied with nutrients.

##### Remark 2

Nutrient availability could indeed explain why plaque development is initiated in the middle of the artery but progresses and manifests in the intima. With a higher energy supply closer to the lumen, cells could be more productive at these sites.

#### Macrophages

A second cell species playing a major role in the formation of atherosclerosis are macrophages. Their behavior is modeled via a diffusion–advection–reaction equation for macrophages concentration $C_{\textrm{Ma}}$

25 $$\begin{aligned}& \dot{C}_{\textrm{Ma}}= {\nabla}_{\textbf{X}}\cdot ( \underbrace{D_{\textrm{Ma}}J \textbf{C}^{-1}{\nabla}_{\textbf{X}}C_{\textrm{Ma}}}_{\text {diffusion}} - \underbrace{r_{\textrm{Ma}}C_{\textrm{Ma}}\textbf{C}^{-1}{\nabla}_{\textbf{X}}C_{\textrm{N}}}_{\text {advection}} )\nonumber \\{} & {} + \underbrace{E_{\textrm{MoMa}}C_{\textrm{Mo}}}_{\text {differentiation}} - \underbrace{E_{\textrm{MaF}}C_{\textrm{Ma}}}_{\text {transformation}} \,. \end{aligned}$$

As monocytes are stimulated to differentiate into macrophages, macrophages accumulate and diffuse through the tissue with $D_{\textrm{Ma}}$ being a diffusion coefficient. When macrophages phagocyte LDL, they transform into foam cells. LDL are not explicitly modeled herein, and it is rather assumed that these are available once inflammation occurred due to the nutrient scarcity. Hence, macrophages transform at a constant rate $E_{\textrm{MaF}}$. Also macrophages are assumed to move preferably toward higher nutrient concentrations, modeled by the advection term with parameter $r_\textrm{Ma}$.

#### Foam cells

Foam cells origin from macrophages that are highly loaded with LDL due to phagocytosis. They are local species and consequently modeled as non-diffusive via a single reaction term

26 $$\begin{aligned} \dot{C}_\textrm{F}= \underbrace{E_{\textrm{MaF}}C_{\textrm{Ma}}}_{\text {transformation}} \,. \end{aligned}$$

This reaction term corresponds to the transformation term of macrophages in Eq. (25).

#### Smooth muscle cells

Smooth muscle cells are attracted by chemokines that are secreted by foam cells. Assuming that the content of these chemokines is proportional to the foam cell content, SMC are modeled to be attracted directly by foam cells. For the sake of simplicity, SMC are considered as local quantities and in one phenotype only. Their evolution equation reads

27 $$\begin{aligned} \dot{C}_\textrm{SMC}= \underbrace{r_\textrm{SMC}\, C_\textrm{F}}_{\text {attraction}} \, \end{aligned}$$

with attraction rate $r_\textrm{SMC}$.

#### Extracellular matrix

Synthetic SMC synthesize collagen. Consequently, collagen production is modeled as a function of SMC

28 $$\begin{aligned} \dot{\rho }_0^\text {c}= r_\text {c}\left( C_\textrm{SMC}- \bar{C}_\textrm{SMC}\right) \left\langle 1 - \frac{\rho _0^\text {c}}{K_\text {c}\bar{\rho }_0^\text {c}} \right\rangle \,. \end{aligned}$$

Herein, $r_\text {c}\ge 0$ is a production parameter that drives collagen synthesis as soon as the SMC concentration increases from the initial concentration $\bar{C}_\textrm{SMC}$, what is considered as accumulation of synthetic SMC. The Macaulay bracket in turn prevents unlimited growth. Parameter $K_\text {c}\ge 1$ specifies thereby the maximum collagen content relative to the initial reference density $\bar{\rho }_0^\text {c}$. For instance, $K_\text {c}= 2$ allows collagen production as long as collagen mass is lower than twice the initial amount. Assuming that the mass ratios $\phi ^{\text {c}j}= \bar{\rho }_0^{\text {c}j}/ \bar{\rho }_0^\text {c}$ of the fiber families with respect to the total amount of collagen remain constant, newly produced collagen distributes along already existing fiber directions

29 $$\begin{aligned} \dot{\rho }_0^{\text {c}j}= \phi ^{\text {c}j}\dot{\rho }_0^\text {c}\,. \end{aligned}$$

In contrast to collagen, the matrix is assumed not to be able to change its mass, i.e.,

30 $$\begin{aligned} \dot{\rho }_0^\text {m}= 0 \,. \end{aligned}$$

This is motivated by the fact that it consists essentially of elastin which has a half-life of decades of years.

### Vasa vasorum network

**Fig. 3:**
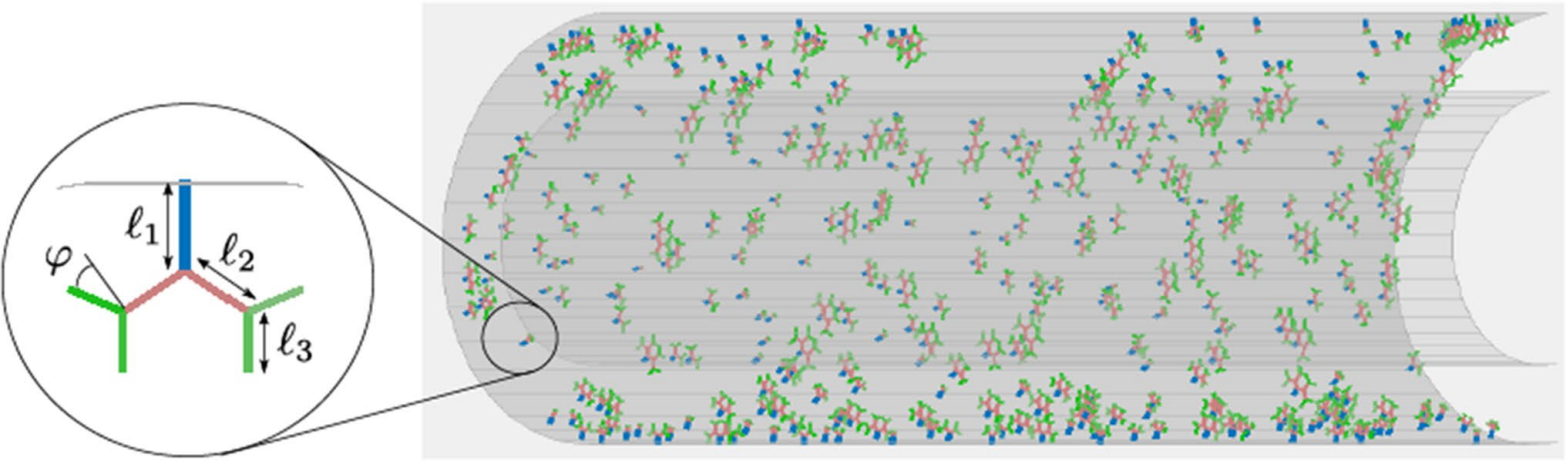
randomly generated vasa vasorum network with characteristic parameters describing the length and orientation of a branch

Arteries are supplied with nutrients from the blood stream and from outside through the vasa vasorum network. The structure and position of the microvessels are herein approximated by randomly generated fractal trees. The code for the generation of the fractal trees is an extended version of the open-source MATLAB code by Dmitry (2022). Starting from the outer surface of the adventitia, they reach into the media of the artery, see Fig. 3, and remain in their axial plane. To account for VV obstruction, a zone in the middle is defined that lacks in trees. From a tree and from every branch, two new branches originate. A tree consists of either two or three branch levels and consecutive tree/branch lengths are in the range $\ell _i/\ell _{i+1} \in (0.9,1.1)$, $i=1,2, \dots$. The azimuthal angle is in the range $\varphi \in (2/5 \, \pi , 8/5 \, \pi )$.

## Finite element implementation

The system of coupled differential equations to be solved includes the balance of linear momentum (12), the mass balances (21), (24)–(28) and the governing equation of remodeling kinematics (15). For the time discretization, a backward Euler finite difference scheme is applied. Discretization in space is based on the Bubnov–Galerkin finite element method with hexahedral finite elements and trilinear Lagrangian polynomial ansatz functions which interpolate all field variables.

The discrete set of primary unknowns $\textbf{p}$ in a finite element is comprised of the nodal displacements $\textbf{u}$ and the diffusive species concentrations of nutrients $C_{\textrm{N}}$, monocytes $C_{\textrm{Mo}}$ as well as of macrophages $C_{\textrm{Ma}}$

31 $$\begin{aligned} \textbf{p}= \{\textbf{u},C_{\textrm{N}},C_{\textrm{Mo}},C_{\textrm{Ma}}\}. \end{aligned}$$

To avoid locking in the context of incompressible growth, a mixed formulation is employed, adding pressure *p* and volumetric dilatation $\theta$ to the set of primary unknowns, see, e.g., Wriggers (2008). This pair of work conjugate variables is chosen to be constant in each element. For the local species concentrations of foam cells $C_\textrm{F}$ and smooth muscle cells $C_\textrm{SMC}$ as well as for the total collagen mass $\rho _0^\text {c}$ and collagen remodeling stretches $\lambda _\text {r}^j$, internal variables are introduced at each Gauss point and merged in a vector $\textbf{h}$

32 $$\begin{aligned} \textbf{h}= \{C_\textrm{F}, C_\textrm{SMC},\rho _0^\text {c},\lambda _\text {r}^j \}. \end{aligned}$$

Discretization in time of evolution equations (26)–(28) and (15) yields a set of algebraic equations $\textbf{Q}(\textbf{p},\textbf{h})=\textbf{0}$ at each Gauss point which is solved for the internal variables via the Newton–Raphson scheme.

To find the primary variables $\textbf{p}$, the balance equations (12), (21), (24) and (25) are formulated in terms of a pseudo-potential

33 $$\begin{aligned}{} & {} \Pi (\textbf{p},\textbf{h}) = \Pi _\textrm{mech}(\textbf{p},\textbf{h}) + \Pi _\text {N}(\textbf{p},\textbf{h}) \nonumber \\{} & {} + \Pi _\textrm{Mo}(\textbf{p},\textbf{h}) + \Pi _\textrm{Ma}(\textbf{p},\textbf{h}) \,. \end{aligned}$$

The variation of this pseudo-potential yields under certain restrictions of internal terms a form that is equivalent to the classical weak formulation of the balance equations, for general details regarding this formulation see Korelc and Wriggers (2016).

The mechanical part of the potential reads

34 $$\begin{aligned}{} & {} \Pi _\textrm{mech}(\textbf{p},\textbf{h}) = \int _{\Omega _0} \left[ \rho _0^{\text {m}} W^{\text {m}}(\textbf{u}) + \sum _{j=1}^{n_\textrm{F}} \rho _{0}^{\text {c}j} W^{\text {c}j}(\textbf{u})\right. \nonumber \\{} & {} \left. + \rho _0 \left( \frac{\kappa }{2} \left( \theta - \frac{\rho _0}{\rho _0(0)} \right) ^{\! 2} + p \, (J-\theta ) \right) \right] \textrm{d}V \,, \end{aligned}$$

with $W^{\text {m}}$, $W^{\text {c}j}$ from Eq. (9).

The pseudo-potential for nutrients is defined as

35 $$\begin{aligned} \Pi _\text {N}(\textbf{p},\textbf{h}) = k_{\textrm{cm}_\text {N}} \int _{\Omega _0} \Bigg [ C_{\textrm{N}}R_\text {N}- {\nabla}_{\textbf{X}}C_{\textrm{N}}\cdot \underbrace{ \left( D_{\text {N}}J \textbf{C}^{-1}{\nabla}_{\textbf{X}}C_{\textrm{N}}\right) }_{=:\textbf{A}_\text {N}}\Bigg ] \textrm{d}V \,, \end{aligned}$$

where the parameter $k_{\textrm{cm}_\text {N}}= 1~\mathrm {m^5 /(kg \, s )}$ is introduced to account for the consistency of units in the potential.

The pseudo-potential for monocytes reads

36 $$\begin{aligned}& \Pi _\textrm{Mo}(\textbf{p},\textbf{h}) = k_{\textrm{cm}_\textrm{Mo}} \int _{\Omega _0} \Bigg [ C_{\textrm{Mo}}\underbrace{ \left( \dot{C}_{\textrm{Mo}}+ E_{\textrm{MoMa}}C_{\textrm{Mo}}+ a_{\textrm{Mo}}C_{\textrm{Mo}}\right) }_{=:A_\textrm{Mo}} \nonumber \\{} & {} - {\nabla}_{\textbf{X}}C_{\textrm{Mo}}\cdot \underbrace{ \left( D_{\textrm{Mo}}J \textbf{C}^{-1}{\nabla}_{\textbf{X}}C_{\textrm{Mo}}- r_{\textrm{Mo}}C_{\textrm{Mo}}\textbf{C}^{-1}{\nabla}_{\textbf{X}}C_{\textrm{N}}\right) }_{=:\textbf{A}_\textrm{Mo}} \Bigg ] \textrm{d}V \,, \end{aligned}$$

with $$k_{\textrm{cm}_\textrm{Mo}} = 1~\mathrm {kg \, m^5 / s }$$.

The pseudo-potential for macrophages is defined as

37 $$\begin{aligned} & \Pi _\textrm{Ma}(\textbf{p},\textbf{h}) = k_{\textrm{cm}_\textrm{Ma}} \int _{\Omega _0} \Bigg [ C_{\textrm{Ma}}\underbrace{ \left( \dot{C}_{\textrm{Ma}}- E_{\textrm{MoMa}}C_{\textrm{Mo}}+ E_{\textrm{MaF}}C_{\textrm{Ma}}\right) }_{=:A_\textrm{Ma}} \nonumber \\ & - {\nabla}_{\textbf{X}}C_{\textrm{Ma}}\cdot \underbrace{ \left( D_{\textrm{Ma}}J \textbf{C}^{-1}{\nabla}_{\textbf{X}}C_{\textrm{Ma}}- r_{\textrm{Ma}}C_{\textrm{Ma}}\textbf{C}^{-1}{\nabla}_{\textbf{X}}C_{\textrm{N}}\right) }_{=:\textbf{A}_\textrm{Ma}} \Bigg ] \textrm{d}V \,, \end{aligned}$$

with $$k_{\textrm{cm}_\textrm{Ma}} = k_{\textrm{cm}_\textrm{Mo}}$$.

The pseudo-potential defined in (33) leads to a nonlinear system of algebraic equations $\textbf{R}= 0$ when spatial and temporal discretization schemes are employed. The associated residual follows by differentiation

38 $$\begin{aligned} \textbf{R} = \textbf{R}(\textbf{p}, \textbf{h}) = \left. \frac{\partial \Pi }{\partial \textbf{p}}\right| _{R_\text {N}, \textbf{A}_\text {N}, A_\textrm{Mo}, \textbf{A}_\textrm{Mo}, A_\textrm{Ma}, \textbf{A}_\text {N}, \textbf{h} = \text {const.}} \end{aligned}$$

where all integrals in (33) are approximated using a Gauss quadrature. The terms that have to be held constant are the internal variables $\textbf{h}$, the reaction term $R_N$ and the terms defined in (35) to (37).

The solution $\textbf{p}$ is obtained employing the Newton–Raphson method

39 $$\begin{aligned} \textbf{p} = \textbf{p}^k - \textbf{K}^{-1} \textbf{R} \!\left( \textbf{p}^k \right) \,, \end{aligned}$$

where superscript *k* indicates variables from the last iteration step. The tangent matrix $\textbf{K}$ results from the consistent linearization of $\textbf{R}$

40 $$\begin{aligned} \textbf{K} = \frac{\textrm{d} \textbf{R}}{\textrm{d} \textbf{p}} = \frac{\partial \textbf{R}}{\partial \textbf{p}} + \frac{\partial \textbf{R}}{\partial \textbf{h}}\frac{\textrm{d} \textbf{h}}{\textrm{d} \textbf{p}} \,, \end{aligned}$$

with $$\textrm{d} \textbf{h}/\textrm{d} \textbf{p}$$ from

41 $$\begin{aligned}{} & {} \frac{\textrm{d}{} \textbf{Q}}{\textrm{d}{} \textbf{p}} = \frac{\partial \textbf{Q}}{\partial \textbf{p}} + \frac{\partial \textbf{Q}}{\partial \textbf{h}}\frac{\textrm{d} \textbf{h}}{\textrm{d} \textbf{p}}=\textbf{0} \nonumber \\{} & {} \quad \Rightarrow \quad \frac{\textrm{d} \textbf{h}}{\textrm{d} \textbf{p}} = - \left( \frac{\partial \textbf{Q}}{\partial \textbf{h}}\right) ^{-1} \frac{\partial \textbf{Q}}{\partial \textbf{p}} \,. \end{aligned}$$

All derivatives as well as the residual ${\textbf {R}}$ and the tangent matrix ${\textbf {K}}$ were computed using the symbolic differentiation tool *AceGen*, for details see Korelc and Wriggers (2016).

For the sake of an efficient numerical implementation, the concentrations are scaled

42 $$\begin{aligned} \begin{aligned} {\hat{C}}_{\text {N}}&=\frac{C_{\textrm{N}}}{10^{-5} \frac{\text {kg}}{\text {m}^3}}\,, \quad {\hat{C}}_{\textrm{Mo}}= \frac{C_{\textrm{Mo}}}{10^{13} \frac{\text {cells}}{\text {m}^3} }\,, \quad {\hat{C}}_{\textrm{Ma}}= \frac{C_{\textrm{Ma}}}{10^{13} \frac{\text {cells}}{\text {m}^3} } \,, \\ {\hat{C}}_{\textrm{F}}&= \frac{C_\textrm{F}}{10^{13} \frac{\text {cells}}{\text {m}^3} } \,, \quad {\hat{C}}_{\textrm{SMC}}=\frac{C_\textrm{SMC}}{10^{13} \frac{\text {cells}}{\text {m}^3}} \, \quad . \end{aligned} \end{aligned}$$

## Numerical simulation of the plaque development

The section presents the performance of the derived model when applied to the development of atherosclerosis. For this a simplified geometry is selected which, however, contains all the main features that influence the development of atherosclerosis.

**Fig. 4:**
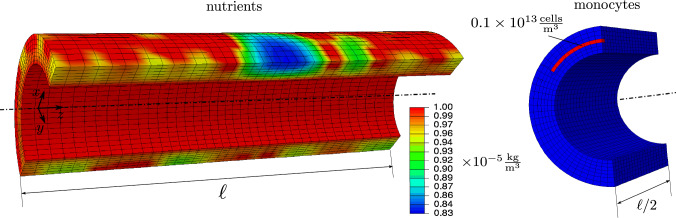
Initial nutrient (left) and monocytes (right) concentrations. Nutrient distribution results from diffusion from lumen and vasa vasorum network, mal-nourishment due to VV obstruction (in blue) causes monocyte accumulation, what is incorporated as Dirichlet boundary condition (in red)

### Geometry, loading and boundary conditions

One-half of an idealized, cylindrical-shaped artery is modeled with inner radius $r_\textrm{i} = 25$ mm, outer radius $r_\textrm{o} = 40$ mm and length $\ell = 200$ mm, see Fig. 4. Symmetry boundary conditions are applied at $y=0$ and the surfaces in the *xy*-plane are fixed in axial direction. To prevent rigid body motion, two nodes in these surfaces at $x=0$ and $r=r_\textrm{i}$ are fixed in *x*-direction. The artery is considered as two-layered tissue, including an adventitia and an intima-media layer with thickness $t_\textrm{A} = 5$ mm and $t_\textrm{IM} = 10$ mm, respectively. Four fiber families are considered ($n_\textrm{F}=4$), two of those oriented along the circumferential and axial direction. The other two families lie in the plane of the former two directions and deviate by $\pm 45^\circ$ from the circumferential direction. The material and simulation parameters are collected in Tables 1 and 2.

Toward a realistic simulation of mechanical conditions in the in vivo configuration, the artery is exposed to a blood pressure $p_{\textrm{blood}}=13$ kPa and is axially stretched by 15 mm. Arteries are often surrounded by fatty or muscular tissues that constrain their movement. Consequently, linear spring elements are added at the nodes of the outer arterial surface to model the embedding by an elastic layer. These springs create a force in radial direction (with a spring stiffness of 80 kN/mm, 160 kN/mm and 320 kN/mm for nodes belonging to one, two and four elements, respectively). Moreover, arteries are initially in homeostasis, being a preferred mechanical state of the tissue. This is incorporated by conducting a preliminary simulation in which the load is applied and held constant such that the artery reaches a stable mechanical state, governed by the elastic prestretch in collagen fibers, cf. Equation (15). The collagen prestretch $\lambda _\text {pre}^j$ is assumed to be identical for all fiber families, $\lambda _\text {pre}^j = \lambda _\text {pre}$. In this preliminary simulation, all concentrations are fixed to their initial values.

With begin of the actual simulation, nutrient supply is incorporated prescribing the nutrient concentration $C_{\textrm{N}}=1. \times 10^{-5}~\mathrm {\frac{kg}{m^3}}$ at the inner surface of the vessel and in elements where VV are present. For the latter nutrient source, finite elements are identified which contain nodes of a vasa vasorum tree. Note that the length of a branch is chosen to be smaller than half the edge length of a finite element to ensure that VV branches have nodes in each element they pass through.

The monocytes recruitment due to nutrient scarcity is modeled as essential boundary condition, prescribing $C_{\textrm{Mo}}= 1. \times 10^{-14}~\mathrm {\frac{cells}{m^3}}$ in the middle region of the media, see Fig. 4.

### Species evolution and plaque growth

**Fig. 5:**
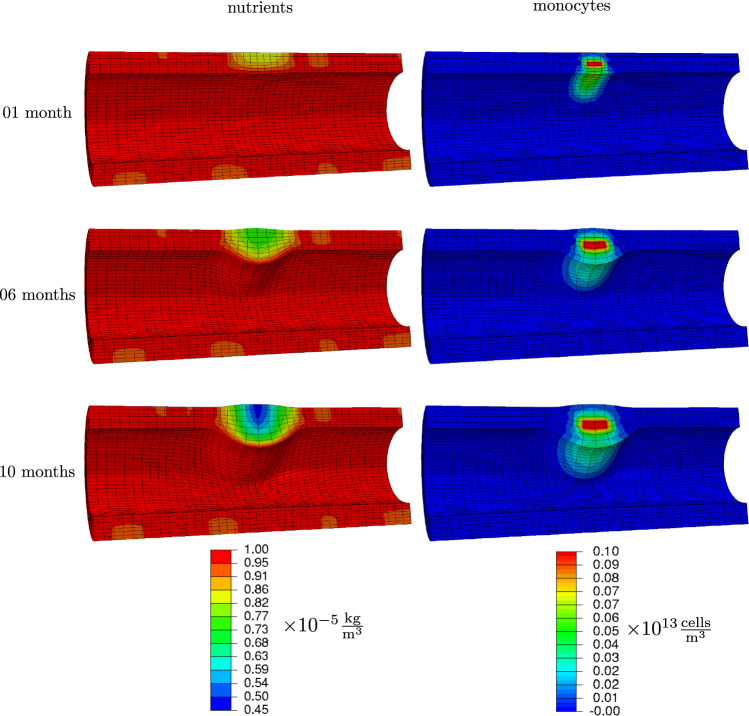
Nutrients and monocytes concentrations 1 month, 6 months and 10 months after occlusion onset

**Fig. 6:**
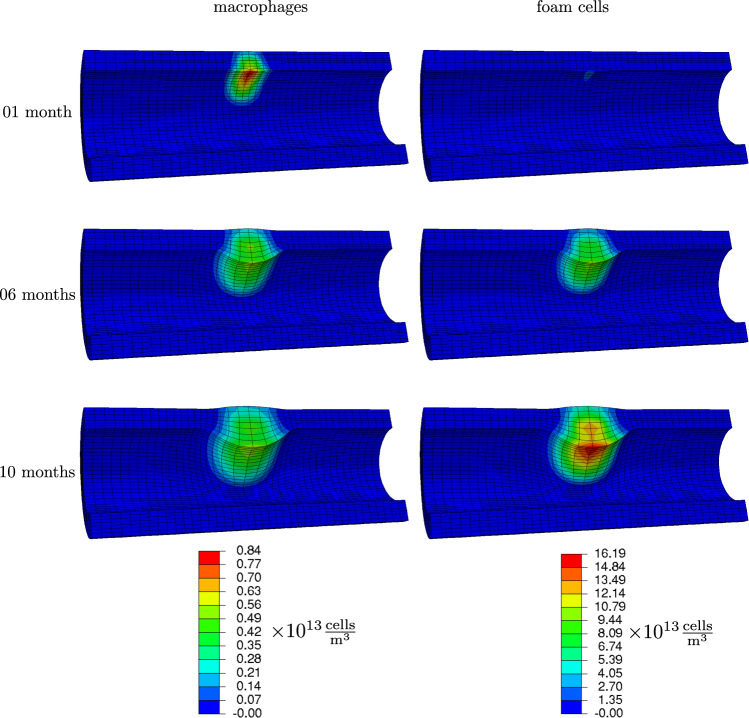
Macrophages and foam cells concentrations 1 month, 6 months and 10 months after occlusion onset

**Fig. 7:**
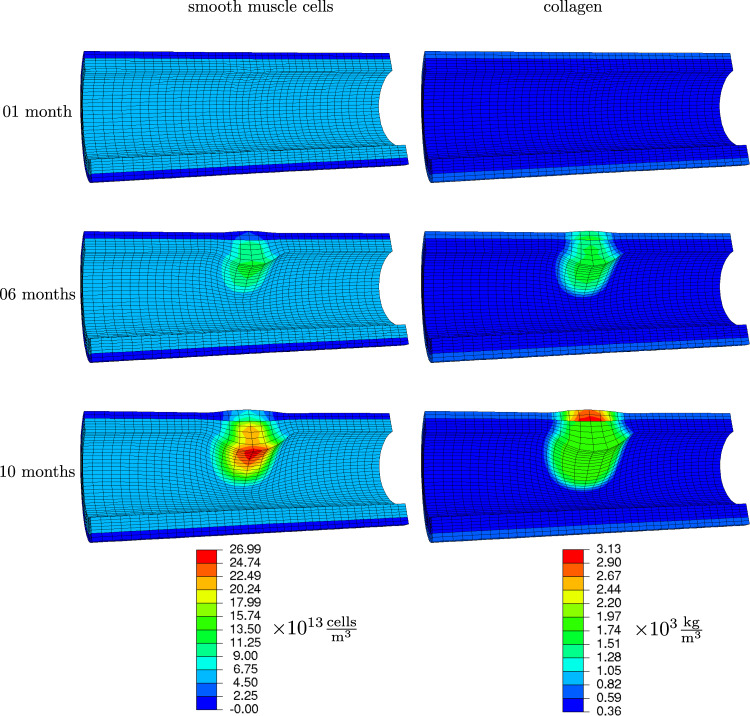
Smooth muscle cells and collagen concentrations 1 month, 6 months and 10 months after occlusion onset

**Fig. 8:**
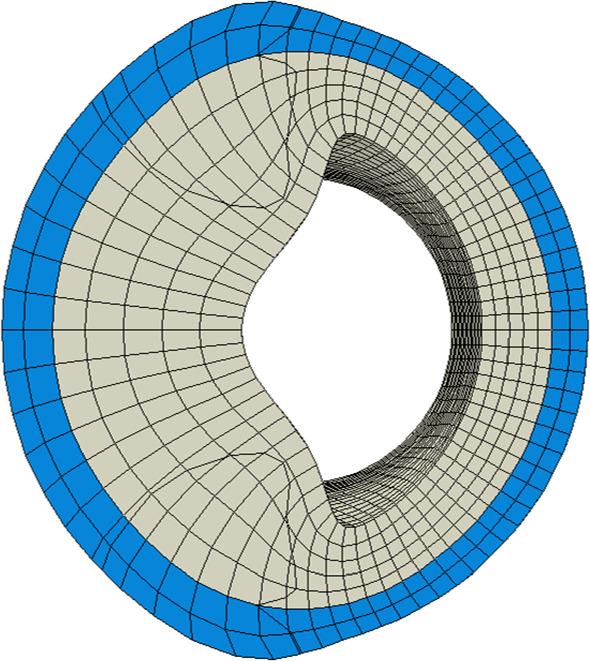
Cross section of arterial wall at $z=\ell /2$ ten months after occlusion onset. The lumen is severely narrowed due to the plaque

The presence of monocytes due to VV obstruction starts the chain of events that lead to plaque growth and hence lumen narrowing. In this example, disease development is tracked one, six and ten months after initial inflammation.

*After one month* Monocytes spread from the center of inflammation, see Fig. 5. Due to diffusion, they are present in a zone around this center. Since they migrate along the nutrient gradient, they accumulated more toward the lumen. From monocytes, macrophages have formed and migrated as well within the tissue, see Fig. 6. This motion was also predominantly along the nutrient gradient such that the macrophages accumulated mainly close to the lumen. Macrophages already started to transform into foam cells, whose concentration is slightly increased close to the lumen.

As a consequence of an increase in monocytes, macrophages and foam cells masses, the artery begins to narrow already after one month. Smooth muscle cells and collagen contents are not yet noticeably increased, see Fig. 7.

*After six months* The plaque size has visibly increased, and the lumen is further narrowed. This is mainly due to the transformation of macrophages into foam cells, see Fig. 6. The differentiation from monocytes to macrophages balances with this transformation such that the amplitude of macrophage concentration did not further increase. However, macrophages continued to distribute spatially. The foam cells in turn attracted smooth muscle cells which settled in the plaque region and copiously synthesized collagen, see Fig. 7. In the media-intima layer, the collagen content is already increased by factor five, what is the maximum allowed concentration (set by parameter $K_\text {c}$).

*After ten months* Foam cells and smooth muscle cells make up a great amount of the plaque. The lumen is now drastically narrowed, see Fig. 8, such that the functionality of the cardiovascular system is very likely impaired. Notably, the nutrient availability has further reduced in the plaque region due to a reduced diffusivity, see Fig. 5.

#### Remark 3

Note that the nutrient scarcity as the source of disease has even exacerbated. This could explain why atherosclerosis hardly stabilizes but proceeds with time.

### Limitations

Although the model based on the outside-in theory can explain major steps of atherosclerosis onset and progression, some results are not yet consistent with clinical data (Virmani et al. 2000). This section highlights those deviations and relates them to biological and mechanical mechanisms that are not yet considered in the model.

In the model, smooth muscle cells have a negligible influence on early plaque progression, whereas in reality, SMC are observed to infiltrate the intima already at this stage (Kolodgie et al. 2007). Moreover, SMC are observed to form a functional layer between the lumen and the developing lipid-rich core of the plaque. A reason for these discrepancies could be that mechano-sensing capabilities of the endothelium and of SMC have not been considered in the model. That is, cellular species sense their mechanical environment and react with biochemical signals, eventually leading to such a SMC increase (Seime et al. 2022). This explanation suggests understanding atherosclerosis as combination of outside-in and inside-out mechanisms, cf. Sect. 1.

Looking at the collagen content close to the lumen, plaques consist of a higher content close to the lumen forming a fibrous cap. Since the model limits the collagen content to prevent unlimited growth, the simulation results show a highly increased but constant collagen content in affected intima-media regions.

## Conclusion

For the first time, a fully coupled mechano-chemo-biological model has been proposed for the outside-in theory of atherosclerosis. The related discretizations in space and time lead to a nonlinear equation system which was embedded into a finite element code. The model captures well the inflammation onset due to VV obstruction in the outer regions of the arterial wall and the disease progression in the intima and media. Growth and remodeling kinematics are captured through state-of-the-art approaches in a nonlinear continuum mechanical framework and coupled with important chemo-biological events. Plaque development is hence not merely phenomenologically described but rather a direct consequence of mass increase due to accumulation of cells and ECM constituents. The results support the theory that atherosclerosis is primarily a microvessel disease and manifests only secondarily as disease in mid- and large-sized arteries, where vasa vasorum are existent, while arteries without vasa vasorum decoration rarely develop the disease (Haverich et al. 2019).

In further research, additional chemo-biological events should be taken into account. For instance, lipoproteins play a major role in cell activation and regulation, and also the interplay of growth factors, matrix metalloproteinases and their inhibitors should be considered, cf. Sect. 1. The contributions of endothelial cells in the chain of events are also undoubted and should be incorporated. Furthermore, smooth muscle cells are known to contribute to the stress–strain response of the artery what should be accounted for in the strain energy function. On the one hand, they act passively like collagen and the matrix. On the other hand, in a contractile phenotype, they actively contract and regulate the blood flow (Murtada et al. 2012). In turn, in a synthetic phenotype, SMC are predominantly involved in the synthesis of ECM constituents. Both cell types should hence be considered in future work as individual cell species. Besides collagen, other extracellular matrix constituents are known to contribute to cell homeostasis and activation as well as lipid retention (Tran-Lundmark et al. 2008; Khalil et al. 2004). An important group are proteoglycans, influencing both SMC quiescence and lipid retention. Consequently, the actions of further key players of the ECM should be incorporated into future models.

Another point of improvement is the mechanical behavior of elastin which is assumed to be isotropic herein. Arranged in fibers, there is evidence that also elastin induces anisotropy (Rezakhaniha et al. 2011) and behaves significantly softer in radial direction than in circumferential and axial direction.

Matured plaques consist of necrotic cores, made up of cholesterol-rich cell debris. These mainly result from necrotic and apoptotic cells such as foam cells and SMC and are not explicitly modeled herein. When plaque stability and rupture are of interest, cellular necrosis and apoptosis should be incorporated (Seimon and Tabas 2009).

## Appendix

(See Tables 1 and 2)

**Table 1:** Initial densities and concentrations, and mechanical material parameters

Description	Parameter	Value	Unit
Initial reference mass densities and concentrations			
Total	$\bar{\rho }_0$	1050.0	$\mathrm {\frac{kg}{m^3}}$
Nutrients	$\bar{C}_{\text {N}}$	0	$\mathrm {\frac{kg}{m^3}}$
Monocytes	$\bar{C}_{\textrm{Mo}}$	0	$\mathrm {\frac{cells}{m^3}}$
Macrophages	$\bar{C}_{\textrm{Ma}}$	0	$\mathrm {\frac{cells}{m^3}}$
Foam cells	$\bar{C}_{\textrm{F}}$	0	$\mathrm {\frac{cells}{m^3}}$
Cell mass	$\alpha$	$10^{-11}$	$\textrm{kg}$
Adventitia			
Matrix	${\bar{\rho }}_{0}^{\text {m}}$	420.0	$\mathrm {\frac{kg}{m^3}}$
Total collagen	$\bar{\rho }_0^\text {c}$	630.0	$\mathrm {\frac{kg}{m^3}}$
Collagen circumferentially aligned	${\bar{\rho }}_{0}^{\text {c}\, 0^\circ }$	63.0	$\mathrm {\frac{kg}{m^3}}$
Collagen axially aligned	${\bar{\rho }}_{0}^{\text {c}\, 90^\circ }$	504.0	$\mathrm {\frac{kg}{m^3}}$
Collagen aligned in $\pm 45^\circ$	${\bar{\rho }}_{0}^{\text {c}\, \pm 45^\circ }$	31.5	$\mathrm {\frac{kg}{m^3}}$
Smooth muscle cells	$\bar{C}_\textrm{SMC}$	0	$\mathrm {\frac{cells}{m^3}}$
Intima-media			
Matrix	${\bar{\rho }}_{0}^{\text {m}}$	240.0	$\mathrm {\frac{kg}{m^3}}$
Total collagen	$\bar{\rho }_0^\text {c}$	360.0	$\mathrm {\frac{kg}{m^3}}$
Collagen circumferentially aligned	${\bar{\rho }}_{0}^{\text {c}\, 0^\circ }$	269.8	$\mathrm {\frac{kg}{m^3}}$
Collagen axially aligned	${\bar{\rho }}_{0}^{\text {c}\, 90^\circ }$	54.2	$\mathrm {\frac{kg}{m^3}}$
Collagen aligned in $\pm 45^\circ$	${\bar{\rho }}_{0}^{\text {c}\, \pm 45^\circ }$	18.0	$\mathrm {\frac{kg}{m^3}}$
Smooth muscle cells	$\bar{C}_\textrm{SMC}$	$4.5 \times 10^{13}$	$\mathrm {\frac{cells}{m^3}}$
Mechanical model			
Isotropic parameter	$\mu$	100.0	$\mathrm {\frac{J}{kg}}$
Volumetric parameter	$\kappa$	$10^5$	$\mathrm {\frac{J}{kg}}$
Collagen prestretch	$\lambda _\text {pre}$	1.062	−
Collagen turnover rate	$k_\text {r}$	$10^{-7}$	$\mathrm {\frac{1}{s}}$
Adventitia			
Anisotropic parameter	$k_1$	52.19	$\mathrm {\frac{J}{kg}}$
Anisotropic parameter	$k_2$	11.15	−
Intima-media			
Anisotropic parameter	$k_1$	3.41	$\mathrm {\frac{J}{kg}}$
Anisotropic parameter	$k_2$	7.77	−

**Table 2:** Chemo-biological material parameters

description	Parameter	Value	Unit
Nutrients			
Density threshold ratio for diffusion speed	$\tilde{\rho }_0$	4.	
Minimum diffusion coefficient	$D_{\mathrm {N_{min}}}$	$1. \times 10^{-12}$	$\mathrm {\frac{m^2}{s}}$
Maximum diffusion coefficient	$D_{\mathrm {N_{max}}}$	$1. \times 10^{-11}$	$\mathrm {\frac{m^2}{s}}$
Nutrient consumption	$R_\text {N}$	$2. \times 10^{-13}$	$\mathrm {\frac{kg}{m^3 \, s}}$
Monocytes			
Diffusion coefficient	$D_{\textrm{Mo}}$	$7. \times 10^{-11}$	$\mathrm {\frac{m^2}{s}}$
Attraction by nutrients	$r_{\textrm{Mo}}$	$1. \times 10^{-4}$	$\mathrm {\frac{m^5}{kg \, s}}$
Differentiation toward macrophages	$E_{\textrm{MoMa}}$	$1. \times 10^{-5}$	$\mathrm {\frac{1}{s}}$
Apoptosis	$a_{\textrm{Mo}}$	$1. \times 10^{-10}$	$\mathrm {\frac{1}{s}}$
Macrophages			
Diffusion coefficient	$D_{\textrm{Ma}}$	$1. \times 10^{-11}$	$\mathrm {\frac{m^2}{s}}$
Attraction by nutrients	$r_{\textrm{Ma}}$	$1. \times 10^{-5}$	$\mathrm {\frac{m^5}{kg \, s}}$
Transformation to foam cells	$E_{\textrm{MaF}}$	$1. \times 10^{-6}$	$\mathrm {\frac{1}{s}}$
Smooth muscle cells			
Attraction by foam cells	$r_\textrm{SMC}$	$1. \times 10^{-7}$	$\mathrm {\frac{1}{s}}$
Collagen			
Production	$r_\text {c}$	$1. \times 10^{-17}$	$\mathrm {\frac{kg}{s}}$
Adventitia			
Upper production threshold ratio	$K_\text {c}$	$\frac{5}{630}$	−
Media			
Upper production threshold ratio	$K_\text {c}$	$\frac{5}{360}$	−
